# Posttraumatic stress disorder and the risk of erectile dysfunction: a nationwide cohort study in Taiwan: PTSD and erectile dysfunction

**DOI:** 10.1186/s12991-021-00368-w

**Published:** 2021-09-28

**Authors:** Sheng-Chiang Wang, Wu-Chien Chien, Chi-Hsiang Chung, Nian-Sheng Tzeng, Yia-Ping Liu

**Affiliations:** 1 Department of Psychiatry, Tri-Service General Hospital, National Defense Medical Center, Songshan Branch, Taipei, Taiwan; 2 Department of Psychiatry, School of Medicine, Tri-Service General Hospital, National Defense Medical Center, 325, Section 2, Cheng-Gung Road, Nei-Hu District, Taipei, Taiwan; 3 Laboratory of Cognitive Neuroscience, Departments of Physiology and Biophysics, National Defense Medical Center, 161, Minquan East Road, Neihu District, Taipei, 11490 Taiwan; 4 Graduate Institute of Medical Sciences, National Defense Medical Center, Taipei, Taiwan; 5 Department of Medical Research, Tri-Service General Hospital, National Defense Medical Center, Taipei, Taiwan; 6 School of Public Health, National Defense Medical Center, Taipei, Taiwan; 7 Taiwanese Injury Prevention and Safety Promotion Association, Taipei, Taiwan; 8 Graduate Institute of Life Sciences, National Defense Medical Center, Taipei, Taiwan; 9 Student Counseling Center, National Defense Medical Center, Taipei, Taiwan; 10 Department of Psychiatry, Chen Hsin General Hospital, Taipei, Taiwan

**Keywords:** Posttraumatic stress disorder, Erectile dysfunction, Cohort study, National Health Insurance Research Database

## Abstract

**Background:**

This study aimed to investigate the association between posttraumatic stress disorder and the risk of developing erectile dysfunction.

**Methods:**

In this population-based retrospective cohort study, we used Taiwan’s National Health Insurance Research Database to analyze patients who were newly diagnosed with posttraumatic stress disorder (PTSD) between 2000 and 2013, with a 1:3 ratio by age and index year matched with patients in a non-PTSD comparison group, for the risk of erectile dysfunction.

**Results:**

In total, 5 out of 1079 patients in the PTSD group developed erectile dysfunction, and 3 out of 3237 patients in the non-PTSD group (47.58 vs. 9.03 per 100,000 per person-year) developed erectile dysfunction. The Kaplan–Meier analysis showed that the PTSD cohort had a significantly higher risk of erectile dysfunction (log-rank, *p* < 0.001). The Cox regression analysis revealed that the study subjects were more likely to develop an injury (hazard ratio: 12.898, 95% confidence intervals = 2.453–67.811, *p* = 0.003) after adjusting for age, monthly income, urbanization level, geographic region, and comorbidities. Psychotropic medications used by the patients with PTSD were not associated with the risk of erectile dysfunction.

**Conclusions:**

Patients who suffered from PTSD had a higher risk of developing erectile dysfunction.

**Supplementary Information:**

The online version contains supplementary material available at 10.1186/s12991-021-00368-w.

## Highlights

- Utilizing a nationwide, population-based database, the Longitudinal Health Insurance Database in 2000–2015 in Taiwan, which comprised 2 million people, we conducted a study to clarify the association between posttraumatic stress disorder and erectile dysfunction.
- Posttraumatic stress disorder was associated with the risk of erectile dysfunction.
- Psychotropic medications in the subjects with PTSD were not associated with the risk of erectile dysfunction.

**Supplementary Information:**

The online version contains supplementary material available at 10.1186/s12991-021-00368-w.

## Introduction

As a devastating and debilitating mental illness that occurs after exposure to traumatic events, posttraumatic stress disorder (PTSD) involves a cluster of symptoms, such as intrusion, hyperarousal, avoiding stimuli associated with traumatic events, and negative alterations in cognition and mood [1, 2]. PTSD can also lead to negative impacts on quality of life and functional impairment in various domains, including sexual dysfunction [3–6]. However, erectile dysfunction (ED) in PTSD is usually underreported in clinical practice and has received little attention in PTSD research, especially in Asian countries.

Few studies have investigated the correlation or rates of ED across PTSD populations [7, 8], and almost no literature has addressed the longitudinal effects of sexual dysfunction. Although it is noteworthy that the percentage of sexual dysfunction is astonishingly high in PTSD patients [9, 10], some inconsistent results have been reported regarding relationship between PTSD and sexual dysfunction [11]. For example, previous studies showed that veterans with sexual dysfunction have significantly more severe PTSD symptoms than those without sexual dysfunction [12]; however, one cross-sectional study in Turkey reported no association between lifetime PTSD and ED [13]. A systemic review reported that the prevalence of sexual dysfunction among veterans with PTSD could be between 8 and 89% in different study sample sizes [11]. Hence, there are still many investigations that need to be explored, including different prevalences of PTSD among all countries and inadequate treatment [14, 15], as well as PTSD and sexual dysfunction. Moreover, it has been suggested that comorbid mental and physical illnesses should be considered an alternative explanation of the co-occurrence of sexual dysfunction and PTSD [9, 16], such as anxiety and depression [17]. Co-occurring physical illnesses may also have bidirectional relationships, as sexual dysfunction is linked to nearly every organ system, including cardiovascular illness (e.g., stroke, coronary artery disease, and hypertension), diabetes mellitus, asthma and alcohol-related diseases [18]. Furthermore, prescription medications could represent the underlying mechanism that explains the co-occurrence of ED and PTSD, such as serotonin reuptake inhibitors and benzodiazepines [19, 20].

There are only a few studies and systematic reviews examining the impact of PTSD on ED in the general population of Asian countries. Since the relationship between PTSD and ED remains unclear, we conducted a nationwide population-based cohort study to investigate the association between PTSD and the risk of ED among Taiwanese people. We hypothesized that there is an increased risk of ED after a PTSD diagnosis, and we used the Taiwan National Health Insurance Research Database (NHIRD) to examine whether there is an association between PTSD and ED.

## Methods

### Data sources

The Taiwan National Health Insurance (NHI) program was launched in 1995 to provide a centralized health insurance system for its citizens, and as of 2014 approximately 93% of the nation’s medical care institutions were contracted, with an enrollment rate exceeding 99% of Taiwan’s population [21]. The NHIRD is derived from the Taiwan NHI program, and all claimed data are released by the Bureau of National Health Insurance for research purposes. The NHIRD uses the International Classification of Diseases, 9th Revision, Clinical Modification (ICD-9-CM) codes to record diagnoses [22]. The quality and validity of the NHIRD is adequate, and its data have been used in many published studies [23–25]. In the present study, we used the datasets from the Registry for the 1-million Longitudinal Health Insurance Database (LHID) which included comprehensive outpatient and inpatient information, such as demographic data, dates of clinical visits, diagnostic codes, and details of prescriptions, with regard to nearly 1 million beneficiaries in Taiwan over a 13-year period from the LHID (2000–2013).

### Study design and sampled participants

**Fig. 1:**
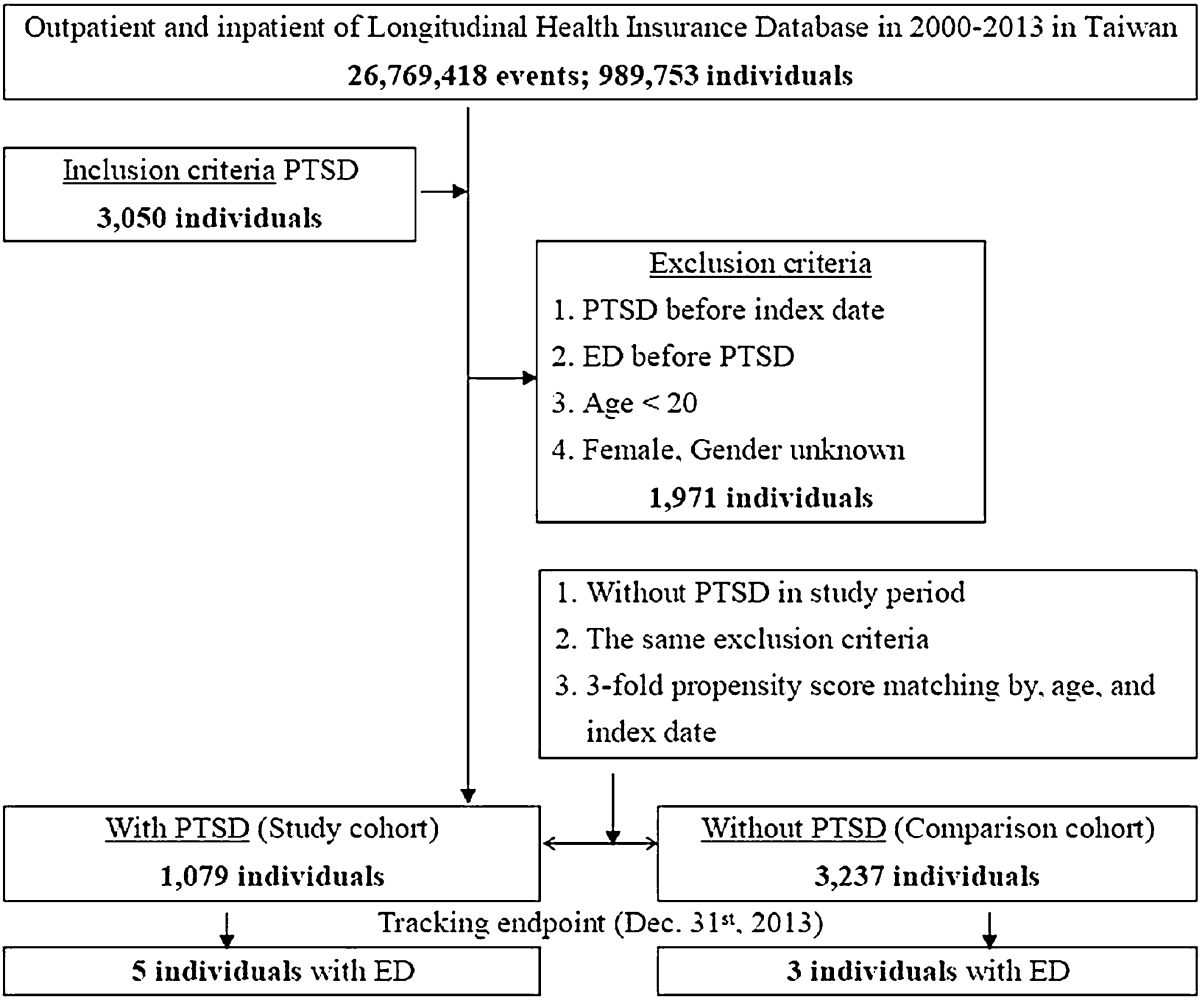
The flowchart of study sample selection

This study was a retrospective study with a matched-cohort design. Patients with PTSD were selected from January 1, 2000, to December 31, 2013, according to ICD-9-CM code 309.81. In addition, each enrolled adult male PTSD patient was required to have made at least three outpatient visits within the one-year study period according to the ICD-9-CM codes. Patients diagnosed with ED before 2000 or before the first visit for PTSD were excluded. In addition, all patients aged < 20 years were excluded. A total of 4310 enrolled patients with 1079 subjects with PTSD and 3237 in the age- and index-year-matched control group without PTSD were included in this study, during a 13-year follow-up to December 31, 2013 (Fig. 1). The required sample size for data analysis is 388 in PTSD group (actual sample size is 1079), and 1164 in non-PTSD group (actual sample size is 3237). Besides, the expected Cohen’s *d* effect size in this study equals to 0.9015. As a sensitivity analysis, by excluding the ED diagnoses within the first and first 5 years after the enrollment of PTSD and non-PTSD groups, we could reduce the protopathic bias or carry-over effects. Therefore, the outcome measures could be more valid.

### Covariates

The covariates included age group (20–39 years, ≥ 40 years), geographic area of residence (north, center, south, west, and east of Taiwan), urbanization level of residence (levels 1–4), levels of hospitals as medical centers, regional hospitals, and local hospitals, and monthly income (in New Taiwan Dollars [NT$]: < 18,000, 18,000–34,999, ≥ 35,000). The Charlson Comorbidity Index (CCI) was used to define comorbidities [26, 27]. The population and various indicators defined the urbanization levels. Urbanization level 1 was defined as a population of > 1,250,000; level 2 was defined as a population between 500,000 and 1,249,999; and levels 3 and 4 were defined as a population between 149,999 and 499,999 and < 149,999, respectively [28].

### Comorbidity

Baseline comorbidities (in ICD-9-CM codes) included dementia, schizophrenia, anxiety disorders, bipolar disorders, depression, stroke, coronary artery diseases (CAD), hypertension, diabetes mellitus (DM), asthma, and alcohol-related illnesses, with the reference from one previous study [29]. All the ICD-9-CM codes of comorbidities were listed in Table S1 (Additional file 1). Data on the usage of psychotropic medications, including antidepressants, antipsychotics, and hypnosedatives, were collected. The defined daily dose (DDD) data were obtained from the WHO Collaborating Centre for Drug Statistics Methodology (https://www.whocc.no/), and the duration of the use of drugs was calculated by dividing the cumulative doses by the DDD of drugs.

### Main outcomes

All of the study participants were followed from the index date until the onset of erectile dysfunction (ED), withdrawal from the NHI program, or the end of 2013. ED was divided into two subgroups: psychogenic ED and organic ED, and the ICD-9-CM codes of ED were listed in Table S1 (Additional file 1).

### Statistical analysis

All statistical analyses were performed using SPSS software V.22 (SPSS Inc., Chicago, Illinois, USA). The *χ*^2^ test and *t*-test were used to evaluate the distributions of the categorical and continuous variables, respectively. Fisher’s exact test was used for the categorical variables to statistically examine the differences between the two cohorts. The Cox regression model was used to determine the risk of psychiatric disorders, and the results are presented as hazard ratios (HRs) with 95% confidence intervals (CIs). The difference in the cumulative incidence of psychiatric disorders between the study and control groups was estimated using the Kaplan–Meier method with the log-rank test. A two-tailed *p* value < 0.05 was considered to indicate statistical significance.

## Results

### Sample characteristics

Table 1 shows that the PTSD group had more anxiety disorders, bipolar disorders, and depression and less CAD and DM than the non-PTSD group. The PTSD group also tended to have lower CCI scores, live in the northern and outlying islands of Taiwan, reside more in urbanization level 2 regions, and receive medical help from medical centers. There were no differences in the distribution of age and insurance premiums between these two groups.

**Table 1:** Characteristics of study at the baseline

PTSD	With		Without		*P*
Variables	*n*	%	*n*	%	
Total	1079	25.00	3237	75.00	
Age (years)	36.05 ± 14.11		36.73 ± 14.28		0.174
Age group (years)					0.999
20–39	869	80.54	2607	80.54	
≥ 40	210	19.46	630	19.46	
Insured premium (NT$)					0.998
< 18,000	967	89.62	2901	89.62	
18,000–34,999	80	7.41	241	7.45	
≥ 35,000	32	2.97	95	2.93	
Dementia	3	0.28	4	0.12	0.337
Schizophrenia	40	3.71	144	4.45	0.338
Anxiety	68	6.30	5	0.15	< 0.001
Bipolar disorder	123	11.40	15	0.46	< 0.001
Depression	471	43.65	24	0.74	< 0.001
Stroke	19	1.76	82	2.53	0.163
Coronary artery disease	7	0.65	97	3.00	< 0.001
Hypertension	49	4.54	165	5.10	0.517
Diabetes mellitus	17	1.58	150	4.63	< 0.001
Asthma	9	0.83	50	1.54	0.095
Alcohol-related disease	49	4.54	118	3.65	0.202
CCI					< 0.001
0	1029	95.37	2794	86.31	
1	25	2.32	231	7.14	
2	11	1.02	70	2.16	
3	6	0.56	93	2.87	
≥ 4	8	0.74	49	1.51	
Antidepressants	959	88.88	246	6.98	< 0.001
1–364 days	311	28.82	157	4.85	
≥ 365 days	648	60.06	69	2.13	
SSRI	892	82.67	153	4.72	< 0.001
1–364 days	345	31.97	104	3.21	
≥ 365 days	547	50.70	49	1.51	
SNRI	937	86.84	136	4.20	< 0.001
1–364 days	338	31.33	99	3.06	
≥ 365 days	599	55.51	37	1.14	
Other antidepressants	948	87.85	162	5.00	< 0.001
1–364 days	375	34.75	112	3.46	
≥ 365 days	573	53.10	50	1.54	
Sedative/hypnotics	884	81.92	173	5.34	< 0.001
1–364 days	431	39.94	95	2.93	
≥ 365 days	453	41.98	78	2.41	
Antipsychotics	839	77.76	137	4.23	< 0.001
1–364 days	368	34.11	66	2.04	
≥ 365 days	471	43.65	71	2.19	
Residence of Taiwan					< 0.001
Northern Taiwan	755	69.97	1315	40.62	
Middle Taiwan	122	11.31	911	28.14	
Southern Taiwan	163	15.11	790	24.41	
Eastern Taiwan	28	2.59	209	6.46	
Outlets islands	11	1.02	12	0.37	
Urbanization level					< 0.001
1 (the highest)	177	16.40	1063	32.84	
2	776	71.92	1362	42.08	
3	57	5.28	289	8.93	
4 (the lowest)	69	6.39	523	16.16	
Levels of hospitals					< 0.001
Medical center	693	64.23	1031	31.85	
Regional hospital	312	28.92	1143	35.31	
Local hospital	74	6.86	1063	32.84	

*PTSD* posttraumatic stress disorder, *P* Chi-square/Fisher exact test on category variables and *t*-test on continue variables, *NT$* New Taiwan Dollars, *CCI* Charlson Comorbidity Index, stroke, coronary artery disease, hypertension, diabetes mellitus and alcohol-related illness, *SSRI* selective serotonin reuptake inhibitor, *SNRI* serotonin norepinephrine reuptake inhibitor

### Kaplan–Meier model for the cumulative risk of erectile dysfunction

**Fig. 2:**
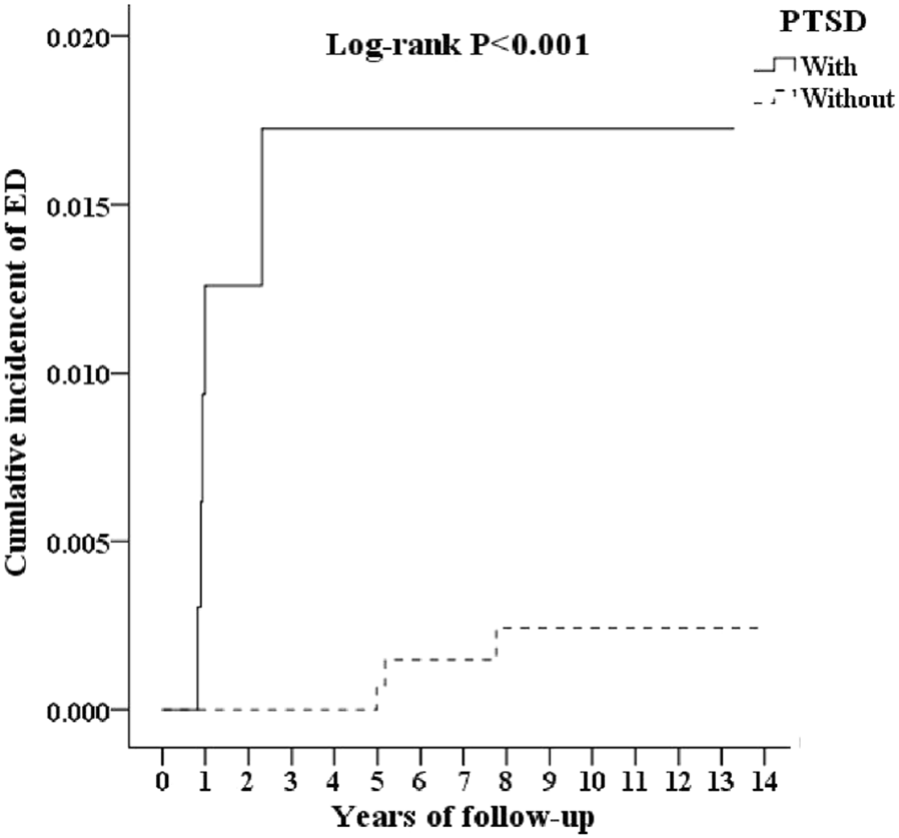
Kaplan–Meier for cumulative incidence of erectile dysfunction aged 20 and over stratified by posttraumatic stress disorder with log-rank test

At the end of the follow-up, five patients in the PTSD group (5 in 1079, 47.58 per 10^5^ person-years) developed ED, and three patients in the non-PTSD group (3 in 3237, 9.03 per 10^5^ person-years) developed ED. The Kaplan–Meier analysis for the cumulative incidence of erectile dysfunction in the study and control groups is shown in Fig. 2 (log-rank test, *p* < 0.001).

### Hazard ratio analysis of ED in patients with PTSD

In the Cox regression analysis model, the crude HR of the PTSD group was 14.766 (95% CI 3.426–63.635, *p* < 0.001), and the adjusted HR of the PTSD group in the development of ED was 12.898 (95% CI 2.453–6–7.811, *p* = 0.003), in comparison to the non-PTSD group, after adjustment for age, insurance premiums, comorbidities, antidepressants, sedatives/hypnotics, urban levels and regions in Taiwan, and the levels of hospitals the patients used for medical care. Patients with comorbidities, such as such anxiety disorders (adjusted HR: 1.864, *p* = 0.025), bipolar disorders (adjusted HR: 1.998, *p* = 0.014), and depression (adjusted HR: 2.970, *p* = 0.001), were associated with the risk of ED (Table 2). The use of antidepressants, antipsychotics, and sedatives/hypnotics by patients were not associated with the development of ED.

**Table 2:** Hazard ratios analysis of ED in the patients with PTSD

Variables	Crude HR	95% CI	95% CI	*P*	Adjusted HR	95% CI	95% CI	*P*
PTSD								
Without	Reference				Reference			
With	14.766	3.426	63.635	**< 0.001**	12.898	2.453	67.811	**0.003**
Anxiety								
Without	Reference				Reference			
With	2.294	1.445	3.978	**0.001**	1.864	1.064	3.568	**0.025**
Bipolar disorder								
Without	Reference				Reference			
With	1.795	1.042	2.568	**0.030**	1.998	1.165	2.774	**0.014**
Depression								
Without	Reference				Reference			
With	4.030	2.457	7.198	**< 0.001**	2.970	1.845	4.798	**0.001**

Adjusted HR: adjusted variables listed in Table 1

*P* Chi-square/Fisher exact test on category variables and *t*-test on continue variables, *PTSD* posttraumatic stress disorder, *HR* hazard ratio, *CI* confidence interval, crude hazard ratio

### Subgroup analysis of ED in the patients with PTSD

Table 3 depicts that PTSD was associated with an increased risk of ED in the PTSD cohort in comparison to the non-PTSD cohort in the subgroup analysis. Compared with patients without anxiety disorders, PTSD had a significantly adjusted HR (7.804, *p* = 0.014). Patients with anxiety disorders and PTSD had a more significant adjusted HR (18.191, *p* < 0.001) than patients with anxiety but not PTSD, with a similar phenomenon in bipolar disorders (8.406 vs. 13.978) and depression (7.975 vs. 19.911).

**Table 3:** Factors of ED stratified by variables listed in the table by using multivariable Cox regression

PTSD	With			Without			With vs*.* without (reference)			
Stratified	Event	PYs	Rate (per 10^5^ PYs)	Event	PYs	Rate (per 10^5^ PYs)	Adjusted HR	95% CI	95% CI	*P*
Total	5	10,508.98	47.58	3	33,224.39	9.03	12.898	2.453	67.811	0.003
Age group (years)										
20–39	3	4533.90	66.17	0	10,458.58	0.00	∞	–	–	0.995
≥ 40	2	5975.08	33.47	3	22,765.80	13.18	6.245	1.198	32.687	0.002
Insured premium (NT$)										
< 18,000	5	10,375.92	48.19	3	32,687.78	9.18	12.898	2.453	67.811	0.003
18,000–34,999	0	129.16	0.00	0	402.26	0.00	–	–	–	–
≥ 35,000	0	3.89	0.00	0	134.34	0.00	–	–	–	–
Dementia										
Without	5	10,474.83	47.73	3	33,104.41	9.06	12.898	2.453	67.811	0.003
With	0	34.15	0.00	0	119.97	0.00	–	–	–	–
Schizophrenia										
Without	5	9605.58	52.05	3	30,821.27	9.73	12.898	2.453	67.811	0.003
With	0	903.40	0.00	0	2403.12	0.00	–	–	–	–
Anxiety										
Without	1	10,327.00	9.68	1	32,922.36	3.04	7.804	1.424	41.027	0.014
With	4	181.98	2198.00	2	302.03	662.19	18.191	3.569	72.774	**< 0.001**
Bipolar disorder										
Without	2	9853.89	20.30	2	32,389.69	6.17	8.046	1.530	42.306	0.007
With	3	655.09	457.95	1	834.70	119.80	13.978	3.121	89.193	**< 0.001**
Depression										
Without	1	9699.42	10.31	1	31,603.57	3.16	7.975	1.517	41.995	0.012
With	4	809.56	494.10	2	1620.82	123.39	19.911	3.697	101.454	**< 0.001**
Stroke										
Without	5	9852.17	50.75	3	31,397.33	9.55	12.898	2.453	67.811	0.003
With	0	656.81	0.00	0	1827.05	0.00	–	–	–	–
Coronary artery disease										
Without	5	9770.07	51.18	2	30,978.06	6.46	18.795	3.685	98.975	0.001
With	0	738.91	0.00	1	2,246.33	44.52	0.000	-	-	0.898
Hypertension										
Without	5	9770.07	51.18	3	30,978.06	9.68	18.795	3.685	98.975	0.001
With	0	738.91	0.00	0	2246.33	0.00	–	–	–	–
Diabetes mellitus										
Without	5	9632.65	51.91	2	28,166.68	7.10	0.000	0.000	0.000	
With	0	876.33	0.00	1	5057.71	19.77	0.000	–	–	
Asthma										
Without	5	10,481.74	47.70	3	32,871.27	9.13	18.795	3.685	98.975	0.001
With	0	27.24	0.00	0	353.12	0.00	–	–	–	–
Alcohol-related diseases										
Without	5	9663.48	51.74	3	31,260.49	9.60	18.795	3.685	98.975	0.001
With	0	845.50	0.00	0	1963.90	0.00	–	–	–	–
CCI_R										
0	4	8437.56	47.41	2	25,127.98	7.96	14.498	2.775	77.045	**< 0.001**
1	1	1208.07	82.78	0	3296.20	0.00	∞	–	–	0.999
2	0	122.52	0.00	1	1097.72	91.10	0.000	–	–	0.897
3	0	403.61	0.00	0	2122.11	0.00	–	–	–	-
≥ 4	0	337.22	0.00	0	1580.38	0.00	–	–	–	–
Antidepressants										
Without	0	1248.79	0.00	1	29,780.20	3.36	0.000	–	–	0.986
1–364 days	2	4054.91	49.32	0	1779.27	0.00	∞	–	–	0.782
≥ 365 days	3	5205.29	57.63	2	1664.92	120.13	1.179	0.223	6.174	0.798
SSRI										
Without	4	1379.70	289.92	2	29,897.01	6.69	106.254	20.174	559.784	< 0.001
1–364 days	0	4103.81	0.00	0	1897.04	0.00	–	–	–	–
≥ 365 days	1	5025.47	19.90	1	1430.33	69.91	0.765	0.124	3.687	0.594
SNRI										
Without	4	1349.01	296.51	2	29,901.12	6.69	108.513	20.634	570.501	< 0.001
1–364 days	1	4098.91	24.40	1	1796.78	55.66	1.073	0.199	5.687	0.751
≥ 365 days	0	5061.06	0.00	0	1526.48	0.00	–	–	–	–
Other antidepressants										
Without	0	1309.10	0.00	2	28,965.87	6.90	0.000	–	–	0.897
1–364 days	3	4077.91	73.57	0	2015.50	0.00	∞	–	–	0.989
≥ 365 days	2	5121.97	39.05	1	2243.01	44.58	2.114	0.408	11.277	0.735
Sedative/hypnotics										
Without	0	1409.78	0.00	1	27,989.45	3.57	0.000	–	–	0.998
1–364 days	2	4813.10	41.55	0	2695.02	0.00	∞	–	–	0.975
≥ 365 days	3	4286.10	69.99	2	2539.92	78.74	2.176	0.414	11.438	0.762
Antipsychotics										
Without	0	1698.91	0.00	1	28,750.12	3.48	0.000	–	–	0.897
1–364 days	2	4340.11	46.08	1	2245.78	44.53	2.533	0.482	13.601	0.513
≥ 365 days	3	4469.96	67.11	1	2228.48	44.87	3.661	0.689	19.265	0.772
Urbanization level										
1 (the highest)	2	3141.06	63.67	2	9110.76	21.95	7.101	1.354	37.301	0.021
2	3	4877.23	61.51	0	14,053.73	0.00	∞	–	–	0.986
3	0	1034.04	0.00	1	3281.11	30.48	0.000	–	–	0.887
4 (the lowest)	0	1456.64	0.00	0	6778.79	0.00	–	–	–	–
Levels of hospitals										
Hospital center	3	3498.68	85.75	2	10,247.28	19.52	10.789	2.049	56.535	**< 0.001**
Regional hospital	2	4968.42	40.25	0	15,631.31	0.00	∞	–	–	0.964
Local hospital	0	2041.88	0.00	1	7345.80	13.61	0.000	–	–	0.997

*NT$* New Taiwan Dollars, *PYs* person-years, *adjusted HR* adjusted hazard ratio: adjusted for the variables listed in Table 1, *P* Chi-square/Fisher exact test on category variables and *t*-test on continue variables, *CI* confidence interval, *CCI_R* Charlson Comorbidity Index, *SSRI* selective serotonin reuptake inhibitor, *SNRI* selective norepinephrine reuptake inhibitor

### Types of ED after PTSD

Table 4 reveals that in the PTSD cohort, PTSD was associated with an increased overall risk of developing ED with an adjusted HR of 12.898 (95% CI 2.453–67.811, *p* = 0.003) and an increased risk of developing psychogenic ED with an adjusted HR of 27.044 (95% CI 2.731–267.795, *p* < 0.001), but not for developing organic ED.

**Table 4:** Factors of erectile dysfunction subgroup by using multivariable Cox regression

PTSD	With vs. without (reference)			
ED subgroup	Adjusted HR	95% CI	95% CI	*P*
Overall	12.898	2.453	67.811	**0.003**
Psychosexual ED (*N* = 6)	27.044	2.731	267.795	**< 0.001**
Organic ED (N = 2)	0.000	–	–	0.998

Adjusted HR: adjusted variables listed in Table 1

*ED* erectile disorder, *PTSD* posttraumatic stress disorder, *HR* hazard ratio, *CI* confidence interval, *P Chi-square/Fisher exact test on category variables and t-test on continue variables.P Chi-square/Fisher* *exact test on category variables and t-test on continue variables.*

## Discussion

Our results supported the study hypothesis that patients with PTSD would have an increased risk of developing erectile dysfunction. The log-rank of the Cox regression model was significant (*p* = 0.003). The adjusted HR was 12.898 (95% CI 2.453–67.811, *p* = 0.003). When compared with previous research on the association between PTSD and the risk of ED [8, 10, 16], this study focused on longitudinal changes in the general population of an Asian country. Previous nationwide cohort studies in the Taiwan NHIRD have reported that PTSD was associated with obstructive sleep apnea [30], bronchial asthma [31], hypertension, DM, dyslipidemia [32], osteoporosis [33], Parkinson’s disease [34], dementia [35], and epilepsy [36]. To the best of our knowledge, this is the first study on the topic of the association between PTSD and the risk of ED in a nationwide, population-based cohort study in an Asian country.

For the lifetime prevalence of PTSD, one study showed ranges from 1.3 to 12.2%, and the one-year prevalence was 0.2 to 3.8% [37]. Another previous study using the NHIRD found that the one-year incidence of PTSD was 1.1% [23]. In our study, the total incidence within the 13-year follow-up period was approximately 0.31% (3050 in 989,753), and when we excluded the 1971 cases of PTSD that did not meet the enrollment criteria of this study, the incidence was 0.11% (1079/989,753); both of these rates were lower than the findings in the 2014 study by Lin et al. This is likely due to the fact that men are hesitant to discuss their sexual lives with their doctors and do not seek medical help for ED and PTSD, or because doctors do not properly document the diagnoses in medical records using ICD-9 codes. Moreover, the discrepancy between the incidences in these two studies might well be related to the strict criteria we employed in the enrollment of PTSD; that is, each enrolled adult male PTSD patient was required to have made at least three outpatient visits within the 1-year study period according to ICD-9-CM codes 309.81.

In consideration of other psychiatric illnesses, such as anxiety, depression and bipolar disorder, our results showed that these comorbidities also contributed to the risk of developing erectile dysfunction; this is compatible with previous findings with bidirectional mechanisms [16, 38]. The percentage of ED in PTSD group due to comorbid depression, anxiety and bipolar disorders is higher, and the result is also consistent with PTSD had psychogenic ED predominantly, not organic ED [39–41]. Endothelial dysfunction, sexual hormones and inflammation in the neural circuitry, susceptible to PTSD, may play crucial roles on the pathogenic effects of ED. Furthermore, in clinical practice guidelines, the most common psychotropic medications used by patients with PTSD include selective serotonin reuptake inhibitors (SSRIs), serotonin norepinephrine reuptake inhibitors (SNRIs), other antidepressants, sedative-hypnotics, and antipsychotics [42]. Among these medications, antipsychotics and lithium are often prescribed for the treatment of PTSD patients and are commonly reported to have adverse effects on sexual function [43, 44]. Previous studies have also reported that antidepressants, antipsychotics and benzodiazepines were associated with ED [45, 46]. However, in this study, the usage of psychotropic medications for PTSD was not associated with an increased risk of ED after adjusting for age, comorbidity, and other covariates. Some probable reasons might be considered, such as poor drug adherence and stigmatization. This implies that more studies are needed to clarify the impact of these medications on the risk of ED in patients with PTSD.

There is an enormous amount of evidence indicating a multifactorial etiology for erectile dysfunction, either organic or non-organic, and a complex interaction exists with psychological, interpersonal, social, cultural, physiological, and gender-influenced processes [47, 48]. Several possible reasons could explain the underlying mechanism. PTSD itself can lead to a higher prevalence of erectile dysfunction, and a higher prevalence of comorbidities exists among patients with PTSD [7]. In addition, patients with PTSD are treated with psychotropic drugs, which can cause side effects that could influence their sexual function [49, 50].

Despite recent studies that highlighted the relationship between ED and PTSD, finding an absolute causation and mechanism of treatment for a patient with ED suffering from PTSD is still challenging. The main limitation of this study is that the number of ED patients in this sample was small, which might be related to underestimation of self-report, stigmatization, and a lower percentage of doctor visits due to cultural factors. Patients with ED may choose not to talk to doctors due to embarrassment, discouragement, or disbelief of the treatment possibilities.

## Conclusions

The patients with PTSD had a higher risk of developing erectile dysfunction than those without PTSD, as determined after adjustment for demographic data and medical and psychiatric comorbidities. PTSD should be considered a predisposing factor in clinical practice while treating patients with erectile dysfunction. As the future direction for research, further study is therefore necessary to clarify the definite pathophysiology between PTSD and erectile dysfunction and to investigate whether prompt interventions for PTSD may reduce ED risk.

## Supplementary Information

**Additional file 1: Table S1.** ICD-9-CM codes of comorbidities and major outcomes.

## Data Availability

The data that support the findings of this study are not openly available due to confidentiality of data collected from the centralized Health and Welfare Data Science Centers.

## Abbreviations

CAD: Coronary artery diseases
CCI: Charlson Comorbidity Index
DDD: Defined daily dose
DM: Diabetes mellitus
ED: Erectile dysfunction
HWDC: Health and Welfare Data Science Centers
ICD-9-CM: International Classification of Diseases, 9th Revision, Clinical Modification
LHID: Longitudinal Health Insurance Database
MOHW: Ministry of Health and Welfare
NHI: National Health Insurance
NHIRD: National Health Insurance Research Database
NT$: New Taiwan Dollars
PTSD: Posttraumatic stress disorder
SNRIs: Serotonin norepinephrine reuptake inhibitors
SSRIs: Selective serotonin reuptake inhibitors
