# Supplementary material for: Posttraumatic stress disorder and the risk of erectile dysfunction: a nationwide cohort study in Taiwan: PTSD and erectile dysfunction
Source: Ann Gen Psychiatry. 2021 Sep 28;20:48. doi: 10.1186/s12991-021-00368-w (PMC8480081; doi:10.1186/s12991-021-00368-w)
Supplement: Supplementary file 1 — Additional file 1: Table S1. ICD-9-CM codes of comorbidities and major outcomes. [file 12991_2021_368_MOESM1_ESM.docx]

| **Table S1. ICD-9-CM codes of comorbidities and major outcomes** | |
| --- | --- |
|  | **ICD-9-CM codes** |
| **Baseline comorbidities** |  |
| **Dementia** | 290, 294.1, 294.2, and 331.0 |
| **Schizophrenia** | 295 |
| **Anxiety Disorder** | 300.0, 300.2, 300.3, 308.3 |
| **Depressive Disorders** | 296.2, 296.3, 311, and 300.4 |
| **Bipolar Disorder** | 296.0, 296.1, 296.4, 296.5, 296.6, 296.7, 296.8, 296.80, and 296.89 |
| **Stroke** | 430–438 |
| **Coronary Artery Diseases** | 410–414 |
| **Hypertension** | 401–405 |
| **Diabetes Mellitus** | 250 |
| **Asthma** | 493 |
| **Alcohol-Related Illness** | 291, 303, 305, 571.0, 571.1, 571.2, 571.3, 790.3, A215, and V11.3 |
| **Major outcomes** |  |
| **Erectile dysfunction** |  |
| **Psychogenic erectile dysfunction** | 302.74 |
| **Organic erectile dysfunction** | 607.84 |

**ICD-9-CM: International Classification of Diseases, Ninth Revision, Clinical Modification**
